# Reproducibility of aortic intima-media thickness in infants using edge-detection software and manual caliper measurements

**DOI:** 10.1186/1476-7120-12-18

**Published:** 2014-06-03

**Authors:** Kate McCloskey, Anne-Louise Ponsonby, John B Carlin, Kim Jachno, Michael Cheung, Michael R Skilton, Jane Koleff, Peter Vuillermin, David Burgner

**Affiliations:** 1 Murdoch Childrens Research Institute, Royal Children’s Hospital, Parkville, Australia; 2 Child Health Research Unit, Barwon Health, Geelong, Australia; 3 University of Melbourne, Parkville, Australia; 4 Deakin University, Burwood, Australia; 5 Boden Institute of Obesity, Nutrition, Exercise and Eating Disorders, University of Sydney, Sydney, Australia

**Keywords:** Aortic intima-media thickness, Edge-detection software, Newborn, Atherosclerosis, Fetal origins of disease

## Abstract

**Background:**

Aortic intima-media thickness measured by transabdominal ultrasound (aIMT) is an intermediate phenotype of cardiovascular risk. We aimed to (1) investigate the reproducibility of aIMT in a population-derived cohort of infants; (2) establish the distribution of aIMT in early infancy; (3) compare measurement by edge-detection software to that by manual sonographic calipers; and (4) assess the effect of individual and environmental variables on image quality.

**Methods:**

Participants were term infants recruited to a population-derived birth cohort study. Transabdominal ultrasound was performed at six weeks of age by one of two trained operators. Thirty participants had ultrasounds performed by both operators on the same day. Data were collected on environmental (infant sleeping, presence of a sibling, use of sucrose, timing during study visit) and individual (post-conception age, weight, gender) variables. Two readers assessed image quality and measured aIMT by edge-detection software and a subset by manual sonographic calipers. Measurements were repeated by the same reader and between readers to obtain intra-observer and inter-observer reliability.

**Results:**

Aortic IMT was measured successfully using edge-detection in 814 infants, and 290 of these infants also had aIMT measured using manual sonographic calipers. The intra-reader intra-class correlation (ICC) (n = 20) was 0.90 (95% CI 0.76, 0.96), mean difference 1.5 μm (95% LOA −39, 59). The between reader ICC using edge-detection (n = 20) was 0.92 (95% CI 0.82, 0.97) mean difference 2 μm (95% LOA −45.0, 49.0) and with manual caliper measurement (n = 290) the ICC was 0.84 (95% CI 0.80, 0.87) mean difference 5 μm (95% LOA −51.8, 61.8). Edge-detection measurements were greater than those from manual sonographic calipers (mean aIMT 618 μm (50) versus mean aIMT 563 μm (49) respectively; p < 0.001, mean difference 44 μm, 95% LOA −54, 142). With the exception of infant crying (p = 0.001), no associations were observed between individual and environmental variables and image quality.

**Conclusion:**

In a population-derived cohort of term infants, aIMT measurement has a high level of intra and inter-reader reproducibility. Measurement of aIMT using edge-detection software gives higher inter-reader ICC than manual sonographic calipers. Image quality is not substantially affected by individual and environmental factors.

## Introduction

Atherosclerosis, the pathological basis of cardiovascular disease, has its origins in the neonatal period [1-3]. Research into the fetal origins of adult-onset cardiovascular disease is increasingly using aortic intima-media thickness as measured by trans-abdominal ultrasound (aIMT) as a putative intermediate phenotype of cardiovascular risk [4]. Aortic IMT has shown to be superior to carotid IMT in both feasibility and association with known cardiovascular risk factors [5,6]. Small exposure-specific studies in newborn infants have reported associations between increased aIMT and cardiovascular risk factors such as intra-uterine growth restriction (IUGR), macrosomia, maternal diabetes, maternal hypercholesterolaemia, and maternal smoking [7-10].

Post mortem studies have shown varying degrees of diffuse intimal thickness present in arteries prone to atherosclerosis from birth [1,11,12]. Ultrasound measurement of intima-media has strongly correlated with histological thickness vessels prone to atherosclerosis, in particular carotid and coronary arteries [13,14]. The reproducibility of newborn aIMT as measured by abdominal ultrasound has only been established in small, tertiary hospital-based studies using specialist vascular ultrasonographers [7,9,10]. Reported intra-class correlations (ICC) between ultrasound readers are high (β = 0.89-0.94) [7,8,15,16]; the largest reported mean difference between sonographers was 16 μm [17]. These results are encouraging, but their generalisability, especially to population-based studies conducted outside the tertiary setting, is uncertain.

To date the only normative data regarding aIMT during early infancy are from two small studies conducted in term infants on days 1–4 of life. These studies used different inclusion criteria and reported substantial differences in mean values and variation in their aIMT measurements [15,16]. Furthermore, these data are different from the distributions presented in other studies of infants at the same age (1–4 days) that compared specific exposure groups (e.g. IUGR infants) to ‘normal’ infants as controls [7,9,10]. Published data only pertain to the first few days of life and the distribution of aortic IMT in older infants has not been reported. Given these limitations, it is relevant to establish the distribution of aIMT in a large, population-derived cohort over the first weeks to months of life. To our knowledge there have been no reported data on the effect of environmental and individual variables on image quality in early infancy or childhood.

This study aimed to establish the reproducibility and distribution of aIMT during early infancy in a birth cohort study conducted in a community setting. In addition it aimed to compare the aIMT measures obtained using edge-detection software and manual sonographic calipers and investigate whether image quality is affected by individual and environmental variables.

## Materials and methods

The Barwon Infant Study (BIS) is a population-derived cohort of 1069 infants recruited prior to 33 weeks gestation. Infants were excluded from BIS if they developed a serious illness in the first few days of life, or if they had major congenital malformations or genetic abnormalities. For the current analysis, preterm infants (<37 weeks gestational age) were excluded.

### Aortic IMT measurement by abdominal ultrasound

Infant aIMT was measured at the 6 week study visit (median age 5.9 weeks [5.1-7.0]). One of two research nurses (operators), trained to perform aortic ultrasounds, conducted the imaging using a GE Vivid I ultrasound machine with a 4–13 MHz linear array vascular transducer. The infant was settled in a quiet room with a parent present. A subset of infants also had a sibling present at the ultrasound. Some infants were given sucrose at operator and parental discretion. Aortic images were obtained in accordance with a standard operating procedure [10,18]. The ultrasound settings were standardised by using presets and images acquired with simultaneous three-lead ECG gating. The abdominal aorta was first identified in cross-section, just above the umbilicus. A longitudinal, straight, unbranched 1 cm segment of abdominal aorta proximal to the abdominal bifurcation was captured between the umbilicus and xiphisternum, using a standard protocol [10,18]. Following identification of both aortic walls, for the assessment of aIMT, the gain and Time Gain Compensation (TGC) settings were used to optimise the image quality. The images were magnified using a resolution box and three continuous cineloops of five or more cardiac cycles were captured and the images stored digitally for off-line analysis.

### Reproducibility, distribution and comparison between measurement methods

**Figure 1:**
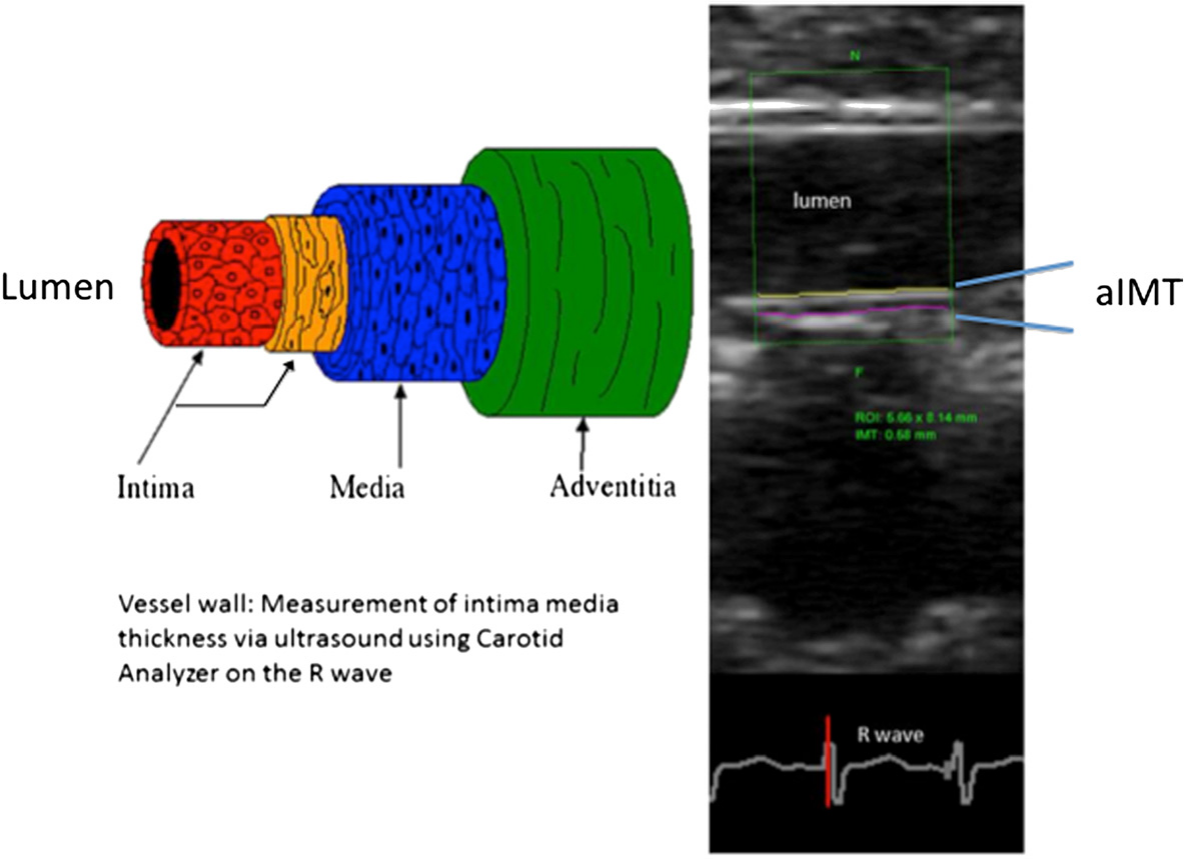
**aIMT measured using edge-detection software, adapted from **[19]**.**

Thirty infants had aortic ultrasounds performed by both ultrasound operators on the same day to allow for measurement of reproducibility between operators.Using edge-detection software (Carotid Analyzer for Research, version 6, Medical Imaging Applications LLC, Iowa), aIMT was measured offline on all infant ultrasounds by one of two readers (who were not ultrasound operators). The best three to five end-diastolic images per infant (timed on the R wave) were analysed. The measurements relied on the edge-detection software identifying the aIMT ‘edge’ along a straight segment of posterior wall, of minimum 4 mm length (Figure 1). The edge-detection software generated a minimum, mean and maximum aIMT for the segment selected. The average of each of these values for an infant’s end-diastolic images was taken as the measure for each infant. In order to calculate the intra-reader (test retest) and inter-reader repeatability of measurements conducted using the edge-detection software, one reader repeated measures in a randomly selected 20 subjects and both readers conducted measures among a separate randomly selected sample of 20 subjects.

In a subset of 290 infants, aIMT was additionally measured using both edge-detection software (as above) and manual sonographic calipers from GE Echopac software. The sonographic caliper measurements were replicated among all 290 infants by two separate readers. Each reader completed 10–20 measures along the aortic wall from consecutive R waves and from at least two cineloops per child to generate an overall mean aIMT. A difference in aIMT of >50 μm on the first measures between readers prompted the aIMT to be re-measured by both readers. If the difference remained >50 μm on repeat measurement, the repeat measurements were taken as final. The average of both readers’ aIMT was taken as the final mean aIMT measurement for each infant. Maximum and minimum aIMT were unable to be measured using this method.

### Individual and environmental variables

The operators recorded the following individual variables; gestation, post-gestational age, age at scan, infant weight, length and head circumference. In addition, environmental variables such as whether the infant was asleep, crying, presence of a sibling, use of sucrose, and timing with other aspects of the overall study protocol were also recorded.

The quality of the aortic cineloops was assessed offline by two readers. Readers were blinded to birth data, individual and environmental variables. Each ultrasound was classified as ‘good’ (both anterior and posterior wall clearly visible throughout the cardiac cycle), ‘adequate’ (posterior wall clearly visible throughout the cardiac cycle) or ‘poor’ (posterior wall intermittently visible, but clearly visible at the time of the R wave). The subsequent reproducibility analyses included all cineloops regardless of this quality classification.

### Statistical methods

The reproducibility (inter-observer variability) of obtaining aIMT measurements between both operators and readers, as well as the intra-reader (test-retest) variability was assessed using ICC and Bland-Altman plots. Associations between the image quality classification and individual and environmental variables were assessed using chi-square test and one-way ANOVA. Statistical analysis was performed using Stata 12.1 (Stata Corp, College Station, TX).

The Barwon Health Human Research Ethics committee approved the study.

## Results

Of the 1069 eligible infants in BIS, 978 (91%) completed their six week visit, during which 844 (86%) had aIMT measured successfully. Excluding preterm infants, aIMT data were available from 814 infants. Five hundred and seventy three of the 814 aIMT images (70%) were assessed as either ‘good’ or ‘adequate’ quality. Gender was evenly represented (53% male), and the average birth weight was 3.5 kg, approximately the 50^th^ centile in the population [20]. In addition, 290 (35%) infants had aIMT measured using sonographic calipers; these infants were similar to the main study cohort (Table 1).

**Table 1:** Comparison of the total cohort of the study and the sub-cohort who had additional measurements of aIMT performed using manual sonographic calipers

**Variables**	**Total Cohort**	Sub-cohort*
	**(n = 814)**	**(n = 290)**
Sex (male)	433 (53%)	150 (51%)
Gestation at birth (weeks)	Mean 39.4 (1.2)	Mean 39.2 (1.2)
Age at time of scan (weeks)	Median 5.9 [5.1-7.0]	Mean 5.6 [4.9-6.1]
Birth weight (kg)	Mean 3.6 (0.5)	Mean 3.6 (0.5)

*Subcohort had aIMT measured using both edge-detection and caliper methods.

### Reproducibility of measurements between operators and readers

**Figure 2:**
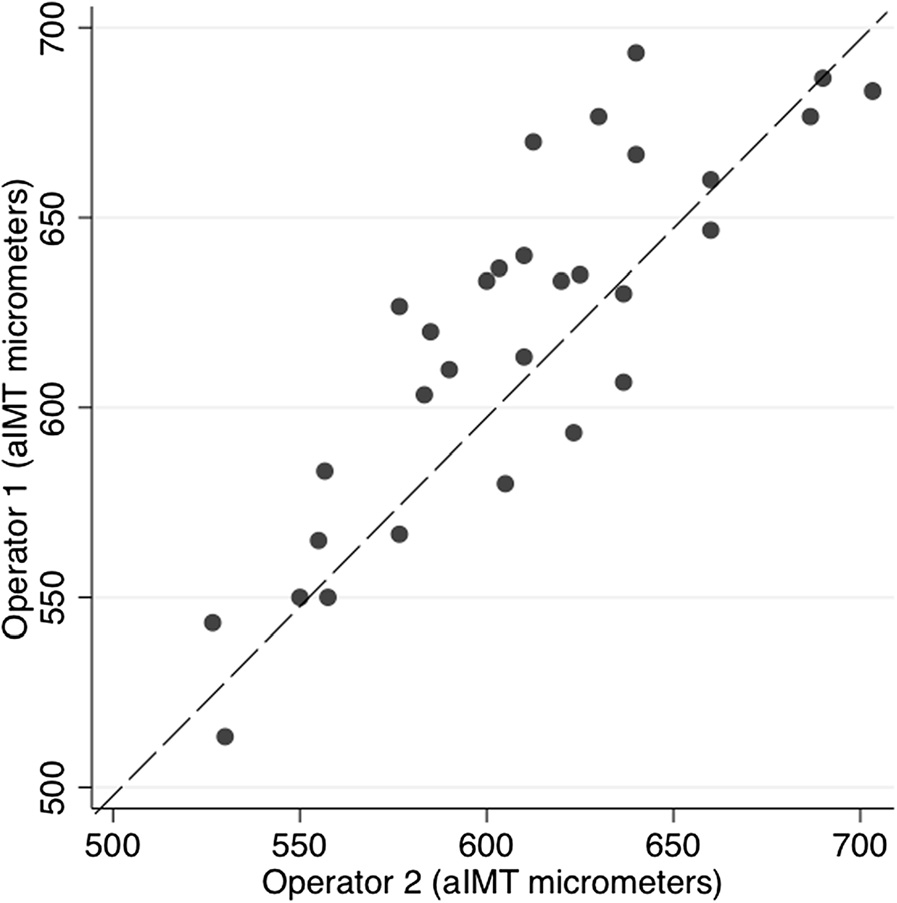
**Comparison of aIMT between ultrasounds performed by separate operators on the same day, using edge-detection software, measured by a single reader.** ICC = 0.84, dashed line y = x.

In the 30 infants who had ultrasounds performed by both operators, the between-operator ICC for the aIMT (measured by a single reader) using edge-detection measurement was 0.84 (95% CI 0.67, 0.92 mean difference 10 μm 95% limits of agreement (LOA) -39, 59), and manual caliper measurement 0.87 (95% CI 0.73, 0.94 mean difference 0.8 μm 95% LOA −50.2, 51.8) (Figure 2).

**Figure 3:**
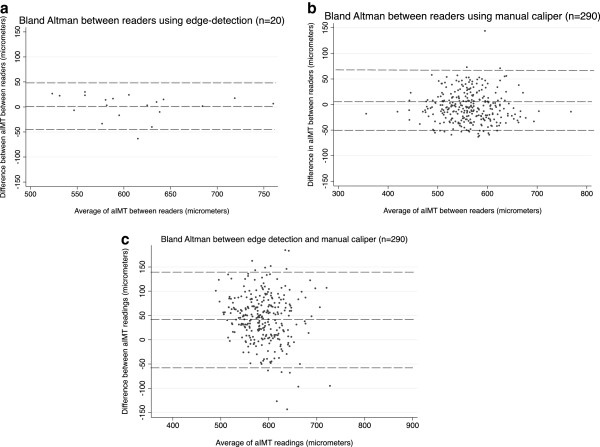
**Bland-Altman plots for agreement between readers in aortic IMT measured by (a) edge-detection (b) sonographic calipers and (c) direct comparison of measurements obtained from edge-detection and caliper methods.** Dashed lines indicate 95% limits of agreement.

Aortic IMT was measured offline by one of two readers using edge-detection software. The intra-reader (test retest) ICC (n = 20) was 0.90 (95% CI 0.76,0.96, mean difference 1.5 μm 95% LOA −45.0, 49). Twenty infants also had aIMT measured by *both* readers. The inter-reader ICC for edge-detection was 0.92 (95% CI 0.82, 0.97, mean difference 2 μm 95% LOA −45.0, 49.0). In addition, aIMT was measured in a subcohort of 290 infants by both readers using manual caliper measures. Of these 290 infants who had their aIMT measured by manual caliper measurement, 42 (14%) had a discrepancy of >50 μm between readers and required repeat measurement. Using the repeat measures as final, the inter-reader ICC for caliper measures was 0.84 (95% CI 0.80-0.87, mean difference 5 μm 95% LOA −51.8, 61.8) (Table 2).Bland Altman analysis of both edge detection versus caliper methods, as well as inter-operator results and inter-reader results using each method, showed no variation between the mean difference and 95% limits of agreement across the spectrum of results (Figure 3).

**Table 2:** Comparison of the intra-class correlation and mean difference for measurements obtained by edge-detection software and manual caliper measurement

	**Measurement method**		
	**Edge detection**	**Manual caliper**	
Inter-operator reliability - *same reader using cineloops from different ultrasound operators*			
n = 30	ICC 0.84 (95% CI 0.67, 0.92)	n = 30	ICC 0.87 (95% CI 0.73, 0.94)
	mean diff 10 μm (95% LOA −39, 59)		mean diff 0.8 μm (95% LOA −50.2, 51.8)
Inter-reader reliability - *two readers using same ultrasound images*			
n = 20	ICC 0.92 (95% CI 0.82, 0.97)	n = 290	ICC 0.84 (95% CI 0.80, 0.87)
	mean diff 2 μm (95% LOA −45.0, 49.0)		mean diff 5 μm (95% LOA −51.8, 61.8)
Intra-reader reliability - *same ultrasound images read twice by the same reader*			
n = 20	ICC 0.90 (95% CI 0.76, 0.96)		N/A (all read by both readers)
	mean diff 1.5 μm (95% LOA −45.5, 48.5)		

### Distribution of aIMT using edge-detection and sonographic caliper techniques

**Figure 4:**
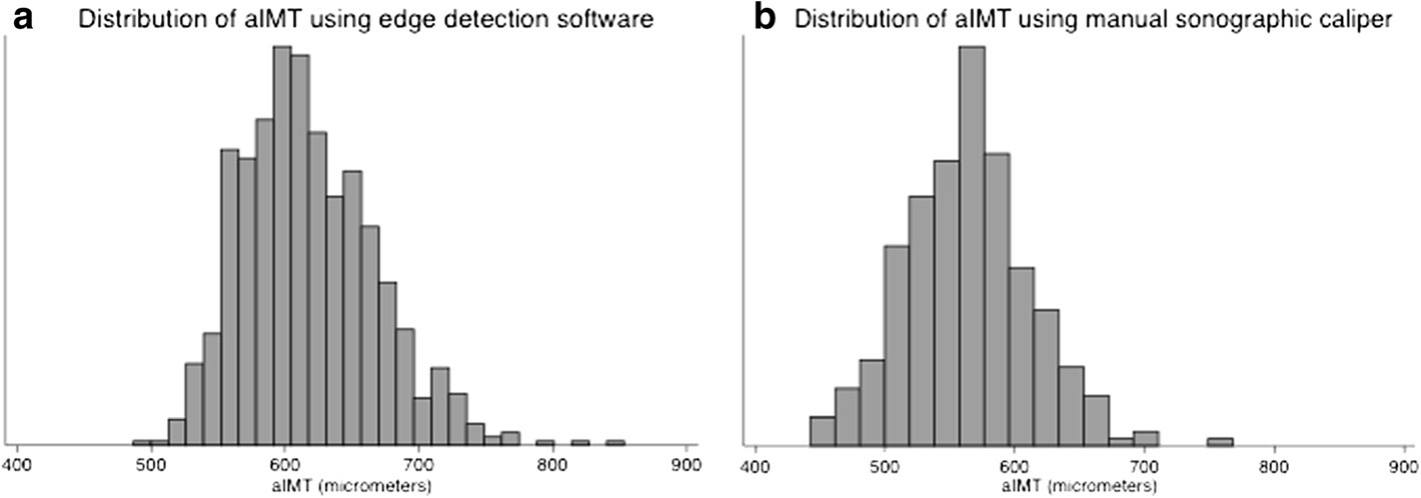
Distribution of aIMT with data obtained using (a) edge-detection mean 618 μm (50) and (b) manual sonographic calipers mean 563 μm (51).

Aortic IMT measurements by edge-detection and manual sonographic calipers each visually approximately followed a normal distribution (Figure 4). The aIMT values obtained using edge-detection were higher (*edge-detection* mean 618 μm (50), *caliper* mean 563 μm, (51) p < 0.001, mean difference 45 μm, 95% LOA −54, 142) (Figure 4).

Aortic IMT measured by edge-detection increased with age at scan (estimated change per week of age 4.82 μm, p < 0.001). Manual sonographic caliper measurement showed a similar association with aIMT, however with borderline significance (change per week of age 4.09 μm, p = 0.074).

### Effect of individual and environmental variables

Infant individual and environmental variables were collected on 500 infants. Infants who were crying were more likely to have poorer images than those who were not (38% versus 23%, p = 0.001). However there was no evidence of an association between other individual and environmental variables and the quality of the images: sleeping (p = 0.11), presence of sibling (p = 0.35), gender (p = 0.45), gestational age at birth (p = 0.84), corrected gestational age at scan (p = 0.24), post-natal age at scan (p = 0.140), birth weight (p = 0.96), timing of the scan in relation to lung function testing (p = 0.34), and sucrose use (p = 0.22).

## Discussion

We have demonstrated that aIMT is reproducible in a large community-based study. There was found high intra and inter-operator ICC between (a) the results obtained using paired ultrasound cineloops obtained by two operators and (b) off-line edge-detection measurements obtained from two readers using the same cineloops, using either edge-detection software or manual caliper measurements. In term infants assessed at 6 weeks, there were differences in aIMT values obtained using different measurement methods; mean aIMT was greater using edge-detection compared to manual sonographic calipers. Furthermore, there was no need for repeat measurements using edge-detection software. With the exception of infant crying, individual and environmental factors did not impact on the quality of images obtained. There was a trend of association between increasing age at scan and higher aIMT measurement.

Previous studies have demonstrated that aIMT is a reproducible measure when conducted by specialist sonographers in a tertiary setting. We have extended these findings by demonstrating that a high reproducibility can be obtained by general research staff following a period of training in the technique. The ICC between research operators performing repeated ultrasounds and readers measuring the same image are consistent with the ICC reported by other groups [7,9,10,16]. Indeed the reproducibility of aIMT measures observed is similar to that reported for adult carotid IMT, an integral part of cardiovascular risk assessment [4]. These findings have important feasibility implications for future population-based research investigating the early life origins of cardiovascular disease.

Our study was able to assess reproducibility but not the dimension of predictive validity –that is we are unable to compare our results to “true” histological measurements of the newborn aorta. We must use intra-observer (test-retest) and inter-observer variability as a proxy for accuracy. A recent study looking at porcine histology as well as aIMT in young children suggested that the use of very-high frequency ultrasound (25–30 Hz) in infants and young children produced more accurate results than high frequency ultrasound may produce more accurate results than high frequency ultrasound, as used in this study [21].Introducing another method of assessment with undoubtedly make comparisons between study results even more difficult.

This study provides the first normal data for healthy infant aIMT data for 6 weeks of age. Values described as ‘normal’ for newborns in the first days of life have been obtained from small studies (n < 100) that have used selected sampling frames and varying definitions of ‘normal’ that are unlikely to be fully representative of the population. For example, a study by Hondappanavar et al. measured aIMT in 100 term infants, but excluded all those below the 50^th^ centile for birth weight [16]. Another study by Koklu et al. investigated preterm and term infants of greater than 24 weeks gestational age, including 60 term infants [15]. This study excluded any infants with putative risk factors for increased aIMT (congenital abnormality, IUGR, macrosomia, maternal smoking, abnormal lipid profiles, maternal dyslipidaemia, hypertension or maternal history of cardiovascular disease) [15]. In contrast, our study was more inclusive; only preterm infants and those with congenital abnormalities or significant neonatal illness were excluded.

In addition to sampling considerations, reported newborn values of mean aIMT are highly variable. Variability may arise due to differences in ultrasound equipment, transducer frequency, and, as our study demonstrates, measurement methods. Using manual sonographic calipers, the following studies reported substantial variation. Whilst Koklu et al. (n = 60) reported a mean of 385 μm with SD 19 at term [15], Hondappanavar et al. (n = 100) found a mean 510 μm (41) [16]. Other data are available from healthy control groups in exposure-specific studies that again used sonographic calipers for measurement. For example Koklu et al. reported, in two separate exposure-specific studies, control values of 400 μm (30) (n = 40) [9] and 390 μm (30) (n = 30) [7]. In contrast Skilton et al. (n = 25) [10] used edge-detection software and reported control values of 534 μm (58) [10]. Surprisingly, the highest control aIMT measures are from Zanardo’s *in utero* study (n = 21) of infants at 32 weeks gestation which reported mean 1050 μm (190) [22]. Zanardo et al. used caliper measurement, but a lower frequency transducer to other studies (3.5-5 Hz versus 8-13 MHz/3-12 MHz). Clearly there are also other significant differences with antenatal imaging such as the depth of imaging, angle of insonation and attenuation that will alter the resolution of images. Our data demonstrate important limitations when comparing results between studies that are not obtained using identical methods. Furthermore, similar to adult studies [23], we show that edge-detection software is at least equivalent to manual caliper measurement in reproducibility (higher ICC) and superior in ease of measurement. Ideally, all future studies would adopt the same ultrasound and measurement protocol to allow accurate comparisons between study results.

In contrast to previous studies that examined infants aged 0–4 days, our study participants varied in age around a median of 5.9 weeks [5.1-7.0]. Aortic IMT has previously been reported to increase with both gestation [15] and age [24]. Concordantly, we found a positive association between aIMT measured by edge-detection software and age at time of scan. Thus the potential influence of gestation and age should be considered when investigating the determinants of aIMT during early life and analyses should be adjusted accordingly.

With the exception of infant crying, we found no evidence that individual and environmental variables, including timing of ultrasound scans during study visits, affected image quality. The effect of environmental variables on image quality is particularly relevant beyond the first days of life, as infants become progressively increasingly more physically active, potentially affecting the quality of the ultrasound images. Our measures of aIMT occurred in the context of multiple other research procedures, such as anthropometric and lung function measurements, and this is likely to the case for many studies of a similar nature to BIS. These results reinforce the feasibility of newborn aIMT measurement in a community-based study where other measures are being taken during a single visit.

## Conclusion

Aortic IMT is a highly reproducible measure that is suitable for use in population-based studies of cardiovascular health and development. In 6 week old infants, aIMT approximates a normal distribution. Results differ depending on the measurement technique (edge-detection vs manual sonographic calipers), so it is imperative that a uniform technique is employed throughout. Edge-detection software has superior reproducibility to manual sonographic caliper measurements. With the exception of infant crying, there is no evidence that image quality is affected by infant behavioural and environmental variables.

## Abbreviations

IMT: Intima-media thickness; aIMT: Aortic intima-media thickness; cIMT: Carotid intima-media thickness; ICC: Intra-class correlation; 95%LOA: 95% limits of agreement; 95% CI: 95% confidence interval.

## Competing interests

The authors declare that they have no competing interests.

## Authors’ contributions

KM was one of the ultrasound and first author. A-L P was involved in study design and drafting. JC was involved in statistical support and drafting. KJ was involved in statistical support and drafting. MC was involved in study design and drafting. MS was involved in the study protocol, design and drafting. JK was one of the readers and involved teaching ultrasound technique. PV was involved in the overall BIS design, study development, funding and drafting. DB was involved in study design, funding and drafting. All authors read and approved the final manuscript.
